# Intra-arterial Cold Saline Infusion in Stroke: Historical Evolution and Future Prospects

**DOI:** 10.14336/AD.2020.0325

**Published:** 2020-12-01

**Authors:** Longfei Wu, Mitchell Huber, Di Wu, Jian Chen, Ming Li, Yuchuan Ding, Xunming Ji

**Affiliations:** ^1^Department of Neurology and China-America Institute of Neuroscience, Xuanwu Hospital, Capital Medical University, Beijing, China.; ^2^Department of Emergency Medicine, Ascension St. John Hospital, Detroit, MI, USA.; ^3^Department of Neurosurgery, Xuanwu Hospital, Capital Medical University, Beijing, China.; ^4^Department of Neurosurgery, Wayne State University School of Medicine, Detroit, MI, USA.

**Keywords:** stroke, neuroprotection, hypothermia, intra-arterial cold saline infusion, clinical translation

## Abstract

Acute ischemic stroke (AIS) is a perpetual threat to life and functionality due to its high morbidity and mortality. In the past several decades, therapeutic hypothermia has garnered interest as an effective neuroprotective method in the setting of AIS. However, traditional hypothermic methods have been criticized for their low cooling efficiency and side effects. Intra-arterial cold saline infusion (IA-CSI), as a novel hypothermic method, not only minimizes these side effects, but is also perfectly integrated with widely accepted recanalization modalities in AIS, thereby serving as a promising prospect for clinical translation. In this article, we review the historical development of IA-CSI, summarize major studies of IA-CSI in rodents, large animals, and humans to date, and suggest insight into future development prospects in the field of AIS. We hope that this article will provide inspiration for the future application of hypothermia in AIS patients.

Acute ischemic stroke (AIS) is an increasingly prevalent threat to human health [1, 2]. A recent epidemiological review concluded that stroke accounts for nearly 5% of all disability-adjusted life years and 10% of all deaths worldwide [3, 4]. Vascular recanalization via intravenous thrombolysis and mechanical thrombectomy has proven very effective under ideal circumstances, but due to a strict therapeutic window, the vast majority of AIS patients are ineligible for intravenous thrombolysis [5]. The inclusion criteria for mechanical thrombectomy are much more inclusive, but outcomes are suboptimal; less than half of patients achieve functional independence at 90 days post-thrombectomy, and 90-day survival is no different than in control groups [6-9]. Given the paucity of effective, accessible stroke therapies, the neurologic community is in desperate need of novel solutions.

Neuroprotective strategies offer this solution. The utility of neuroprotection has been long-recognized, and thousands of neuroprotective strategies have been evaluated, but among them, therapeutic hypothermia has demonstrated the most promise [10]. The general utility of therapeutic hypothermia dates back to Hippocrates, but modern research on neuroprotective hypothermia began in the 1980’s when the Busto group identified the importance of brain temperature in neuronal preservation following periods of transient global ischemia [11, 12]. In the subsequent decades, the neuroprotective mechanisms of therapeutic hypothermia have gradually elucidated, including its effects on cerebral blood flow, metabolism, apoptosis, inflammation, blood-brain barrier integrity, angiogenesis, neurogenesis, and gliogenesis [13]. Therapeutic hypothermia interferes with multiple cell-death pathways over the acute, subacute, and chronic phases of brain injury [13].

Hypothermia is a well-established therapy in the fields of cardiac arrest and neonatal hypoxic-ischemic encephalopathy. Two randomized controlled trials demonstrated almost simultaneously that patients with cardiac arrest could benefit from therapeutic hypothermia [14, 15]. The subsequent Targeted Temperature Management (TTM) trial, which explored the optimal therapeutic temperature for hypothermia, was inconclusive, but based on the successes of the initial trials, therapeutic hypothermia remains a standard of care for out of hospital cardiac arrest [16, 17]. Therapeutic hypothermia has also been found to reduce the risk of death and major sensorineural disability in newborns with hypoxic-ischemic encephalopathy [18], which is the major evidence behind its recommendation for widespread clinical implementation [17, 19]. However, despite the abundance of encouraging data, hypothermia has never been clinically implemented in the context of AIS.

Systemic cooling, the technique used in cardiac arrest and neonatal hypoxic-ischemic encephalopathy, has been evaluated in AIS with discouraging results. A randomized, multicenter trial of endovascular cooling in AIS patients treated within 6 hours of symptom onset found that 18% of patients receiving hypothermia achieved favorable outcomes (modified Rankin Scale score of 0 or 1) at 90-day follow-up, compared to 24% of patients in the normothermia group [20]. Another trial suggested that both hypothermia and normothermia conferred the same (39%) 90-day functional independence outcomes (modified Rankin Scale score of 0 to 2) [21]. In both studies, however, the systemic effects of hypothermia led to a far greater frequency of adverse events, including shivering, bradycardia, hypertension, congestive heart failure, hyponatremia, hypokalemia, hypomagnesemia, hypoxemia, hypercapnia, and acidosis [22-24]. The Intravenous thrombolysis plus hypothermia for acute treatment of ischemic stroke (ICTuS-L) trial also reported a 50% incidence of pneumonia in the hypothermia group, compared to 10% in the normothermia group [20]. While these detriments may be warranted in exchange for outstanding neuroprotective efficacy, cooling provides diminishing return as ischemic time increases. Traditional surface cooling methods take 3-7 hours to reach target temperatures, placing patients outside the 2-3 hour window in which hypothermia is maximally protective [25].

Since hypothermia in AIS is hindered by systemic effects, an optimal strategy would avoid systemic cooling altogether. To this end, local cooling methods have been devised. Cooling helmets were investigated [26], but target temperatures were difficult to attain through the skull [27]. Endovascular heat exchangers have also been evaluated, but were also unable to achieve acceptable cooling rates [28]. A pair of papers published by Ding, et al. in 2002 offered a solution through the concept of intra-arterial cold saline infusion (IA-CSI) [29, 30]. The IA-CSI concept achieved hypothermia via infusion of ice-cold saline through an endovascular microcatheter guided directly to the infarct site under image guidance [27]. By restricting cooling efforts to the ischemic region, IA-CSI effectively circumvented the side effects of whole-body cooling. Thermodynamic models estimate that IA-CSI can cool 300 g of brain tissue at 1 °C per minute [31], which is 10-20 times faster than endovascular cooling, and 18-42 times faster than the 3-7 hours for surface cooling [32]. Although IA-CSI is invasive, it could easily be performed with only the catheter used for general endovascular procedures. In the current era of reperfusion, IA-CSI complements mechanical thrombectomy, as cold saline could easily be run through the thrombectomy catheter.

In recent years, IA-CSI has made significant progress in the field of AIS. From rodent to large animal studies, then to clinical studies (Table 1), the procedure has shown great promise. This review focuses on the clinical translation of IA-CSI from benchtop to bedside in the context of AIS to lay a foundation for clinical implementation of this promising neuroprotective strategy.

## IA-CSI in rodent studies of stroke

The initial 2002 Ding papers evaluated the cooling efficacy of pre-reperfusion flushing in a rat stoke model [29]. Transient middle cerebral artery occlusion (tMCAO) was achieved using a hollow intraluminal filament. After 2 hours of cerebral ischemia, a total of 7 ml of isotonic saline at 23 °C or 37 °C was infused through the filament into the ischemic region at 2 ml/min. Saline infusion at 23 °C and 37 °C both significantly reduced infarct volumes and improved functional neurologic outcomes at 48 hours after reperfusion [29]. A follow-up rat study again found improved functional outcomes after flushing, and that these benefits extended over a full 28-day postoperative period [33]. Together, these data implied the benefit of pre-reperfusion flushing regardless of temperature [30]. A numerical benefit was additionally appreciated in both infarct volumes and neurologic deficits of rats receiving IA-CSI compared to warm local infusion, but neither study included a large enough sample size to achieve statistical difference. Mechanistically, saline flushing was found to reduce post-reperfusion inflammatory mediator expression in ischemic rats, including IL-1beta, TNF-alpha, and ICAM-1 [34]. Pre-reperfusion infusion also ameliorated cerebral edema and reduced matrix metalloproteinase overexpression, suggesting a component of blood-brain barrier integrity preservation [35]. This blood-brain barrier protection was recently corroborated by the Kurisu group in a 2015 paper, where pre-reperfusion cold saline flushing was also found to inhibit the post-reperfusion acute aquaporin-4 surge, attenuate microvascular narrowing, and decrease inflammatory cascade activation [36].

**Table 1:** Summary of studies on intra-arterial cold saline infusion.

Authors	Subject	Model	Infusate	Infusion rate	Infusion duration	Infusion volume	Time to target temp	Brain temp	Core body temp	Infarct volume	Functional outcome
Rodent studies											
Ding et al., 2002 [29]	Rat	tMCAO	Saline 23 °C	2 ml/min	3-4 min	7 ml	3-4 min	32-33 °C	Not mentioned	Reduced	Improved
			Saline 37 °C	2 ml/min	3-4 min	7 ml	-	37 °C	Not mentioned	Reduced	Improved
Ding et al., 2002 [30]	Rat	tMCAO	Saline and heparin 23 °C	3 ml/min	3-4 min	8-10 ml	4 min	32-33 °C	Unchanged	Reduced	Improved
Ding et al., 2003 [34]	Rat	tMCAO	Saline 37 °C	2 ml/min	3 min	6 ml	-	Not mentioned	Not mentioned	Not mentioned	Not mentioned
Ding et al., 2004 [35]	Rat	tMCAO	Saline 37 °C	2 ml/min	3 min	6 ml	-	Not mentioned	Not mentioned	Not mentioned	Not mentioned
Ding et al., 2004 [33]	Rat	tMCAO	Saline 20 °C	0.6 ml/min	10 min	6 ml	< 5 min	Cortex 33.4 °C Striatum 33.9 °C	> 36 °C	Reduced	Improved
Kurisu et al., 2016 [36]	Rat	tMCAO	Saline 10 °C	0.32-0.41 ml/min	15 min	4.8-6.2 ml	< 5 min	Cortex 34.8 °C Striatum 35.4 °C	> 37 °C	Reduced	Improved
Li et al., 2004 [37]	Rat	tMCAO	Saline 20 °C	0.6 ml/min	10 min	6 ml	Not mentioned	Not mentioned	Not mentioned	Reduced	Improved
Luan et al., 2004 [38]	Rat	tMCAO	Saline 20 °C	0.6 ml/min	10 min	6 ml	< 5 min	Cortex 33.4 °C Striatum 33.9 °C	> 36 °C	Not mentioned	Not mentioned
Zhao et al., 2009 [39]	Rat	tMCAO	Saline 20 °C	0.6 ml/min	10 min	6 ml	< 10 min	Cortex 32.8-33.2 °C Striatum 33.2-33.3 °C	> 37 °C	Reduced	Improved
Ji et al., 2012 [70]	Rat	tMCAO	Saline 10 °C	0.25 ml/min	30 min 10/10/10 min	7.5 ml	6 min	34.6 °C	37 °C	Reduced	Unchanged
Ji et al., 2012 [71]	Rat	tMCAO	Saline 10 °C	0.17-0.42 ml/min	20 min	Not mentioned	Not mentioned	33-34 °C	37 °C	Reduced	Improved
Kurisu et al., 2016 [42]	Rat	pMCAO	Saline 4 °C	0.28-0.37 ml/min	15 min	4.2-5.6 ml	< 5 min	Cortex 32.5 °C Striatum 34.3 °C	> 37 °C	Reduced	Improved
Song et al., 2013 [45]	Rat	tMCAO	Magnesium sulfate 15 °C	0.4 ml/min	20 min	8 ml	5-10 min	33-34 °C	37 °C	Reduced	Improved
Chen et al., 2013 [46]	Rat	tMCAO	Human albumin 0 °C	Not mentioned	Not mentioned	Not mentioned	< 3 min	Cortex 30.5 °C Striatum 30.8 °C	37-37.5 °C	Reduced	Improved
Wu et al., 2017 [47]	Rat	tMCAO	Saline 20 °C plus DHC	0.6 ml/min	10 min	6 min	< 10 min	< 35 °C	35 °C	Reduced	Improved
Wu et al., 2019 [48]	Rat	tMCAO	Saline 4 °C plus DHC	0.6 ml/min	10 min	6 ml	Not mentioned	Not mentioned	Not mentioned	Reduced	Improved
Wei et al., 2019 [49]	Rat	tMCAO	Saline 4 °C plus MSC	0.6 ml/min	5 min	3 ml	Not mentioned	Not mentioned	Not mentioned	Reduced	Improved
Large animal studies											
Furuse et al., 2007 [51]	Canine	-	Ringer’s solution 6.5 °C	38.9-43.4 ml/min	30 min	> 1000 ml	30 min	33.6 °C	34.1 °C	-	-
Wang et al., 2016 [52]	Rhesus monkey	-	Ringer’s solution 0-4 °C	5 ml/min	20 min	100 ml	10 min	Cortex 34 °C Striatum 33.9 °C	37.1 °C	-	-
Mattingly et al., 2016 [57]	Swine	tMCAO	Not mentioned	Not mentioned	36-150 min	Not mentioned	< 30 min	26 °C	34 °C	Reduced	Not mentioned
Caroff et al., 2019 [53]	Canine	-	Saline 4.5 °C	20-40 ml/min	14.4 min	515 ml	< 5 min	23.8 °C	37.2 °C	-	-
		tMCAO	Saline 4.5 °C	22 ml/min	25 min	550 ml	< 5 min	31-32 °C	37.2 °C	Reduced	Improved
Clinical studies											
Choi et al., 2010 [58]	Non-stroke patients	-	Saline 4-17 °C	33 ml/min	10 min	330 ml	< 10 min	-0.84 °C (JVBT)	-0.15 °C	-	-
Chen et al., 2016 [62]	AIS patients	-	Saline 4 °C	10 ml/min (IBR) 30 ml/min (IAR)	5 min (IBR) 10 min (IAR)	350 ml	Not mentioned	-2 °C	-0.1 °C	-	-
Wu et al., 2018 [63]	AIS patients	-	Saline 4 °C	10 ml/min (IBR) 30 ml/min (IAR)	5 min (IBR) 10 min (IAR)	350 ml	Not mentioned	Not mentioned	36.5 °C	Reduced	Unchanged

tMCAO, transient middle cerebral artery occlusion; pMCAO, permanent middle cerebral artery occlusion; DHC, dihydrocapsaicin; MSC, mesenchymal stem cell; JVBT, jugular venous bulb temperature; AIS, acute ischemic stroke; IBR, infusion before reperfusion; IAR, infusion after reperfusion.

Based on the success of pre-reperfusion flushing, the IA-CSI concept was put forth in three consecutive 2004 papers [33, 37, 38]. These studies differed from the pre-reperfusion flushing papers in several ways. Chiefly, infusion was performed simultaneously with reperfusion. They also employed more targeted cooling, and featured a colder (20 °C), slower (0.6 ml/min), and longer (10 min) infusion [33, 37, 38]. Target temperatures (33-34 °C) were achieved in the cortex and striatum within 5 minutes and maintained for up to 60 minutes after reperfusion without clinically significant vital sign aberrations [33, 38]. Furthermore, IA-CSI led to marked reductions in infarct volumes and improvements in neurofunctional preservation compared to systemic hypothermia or local saline infusion at body temperature (37 °C) [33, 37]. A follow-up tMCAO rat study found that IA-CSI extends the window of maximal recanalization efficacy from 2 to 2.5 hours. As such, a combination IA-CSI/recanalization strategy could likely broaden the therapeutic window for recanalization in AIS patients [39].

Post-reperfusion flushing, a logical extension of pre-reperfusion flushing, has also demonstrated a significant neuroprotective effect with increased clinical relevance, since recanalization is of primary importance in care of the ischemic stroke patient. A 2012 study by Ji and colleagues used two methods to infuse cold saline after recanalization: the traditional continuous infusion method (uninterrupted IA-CSI), and infusion with 3 20 minute interruptions interspersed at regular intervals (interrupted IA-CSI) [40]. While both methods significantly reduced infarct volume and cerebral edema in a rat model, interrupted IA-CSI considerably extended hypothermic duration without hemodilution. A follow up study by the same group evaluated IA-CSI or intra-carotid body-temperature saline infusion with variable post-reperfusion latency periods [41]. At smaller post-reperfusion latency periods, infarct volumes decreased independent of infusion temperature, although IA-CSI led to lower brain water content, lower cell death marker expression, and improved neurologic function. With larger post-reperfusion latency periods, only IA-CSI displayed efficacy, implying that cold infusion broadens the therapeutic window for infusion [41]. Given the clinical relevance of post-reperfusion flushing, these investigations have served as the basis for numerous subsequent large animal and human trials.

In addition to the tMCAO model, the IA-CSI concept has also demonstrated efficacy in a permanent MCAO rat model. Using immunohistological analysis, Kurisu and colleagues observed suppression of apoptosis and reactive gliosis in the ischemic penumbra of rats, despite permanent MCA occlusion [42]. A sizable proportion of stroke patients do not achieve recanalization, so the efficacy of IA-CSI may provide a therapeutic option regardless recanalization success [43].

IA-CSI has also been found to augment the efficacy of other neuroprotective agents [44]. In 2013, Song and colleagues infused a hypothermic magnesium sulfate solution (15 °C) into the internal carotid artery of ischemic rats. Compared to infusion of saline at the same temperature, the hypothermic magnesium sulfate solution further reduced the infarct volume, brain water content, and attenuated neurologic deficits [45]. The neuroprotective effects of local cold saline infusion (0 °C), local cold human albumin infusion (0 °C), local normothermic human albumin infusion (37 °C), and systemic normothermic human albumin infusion (37 °C) have also been compared in a tMCAO rat model, with local cold human albumin infusion (0 °C) conferring the smallest infarct volumes and best functional outcomes [46]. Synergistic efficacy has also been observed in IA-CSI coadministration with the pharmacologic cooling agent dihydrocapsaicin. In a pre-clinical study, Wu and his colleagues found that, although an acceptable cooling and neuroprotective effect could be achieved with IA-CSI or dihydrocapsaicin alone, their combined administration further lowered brain temperature, reduced infarct volumes, and ameliorated neurologic deficits in ischemic rats. The combination of physical and pharmacologic hypothermia positively affected energy metabolism, oxidative stress, cell apoptosis, blood-brain barrier integrity, and inflammatory responses [47, 48]. Stem cell therapy also benefits from IA-CSI, as mesenchymal stem cell exert more profound neuroprotective effects when combined with IA-CSI [49]. The underlying mechanism is related to the stronger anti-inflammatory and anti-apoptotic effects of the pairing. In vitro experiments also revealed that Miro1, a mitochondrial transfer protein implicated in improved neurologic recovery, was significantly upregulated in stem cells at low temperature [49].

While IA-CSI provides robust neuroprotective benefits in rodents, findings in rat models often translate poorly to human trials [50]. In addition to the well-known species differences between rodents and humans, differences in brain volume may also determine the success of clinical translation, since smaller brains likely cool more quickly. As such, large animal studies were a necessary requisite for clinical implementation.

## IA-CSI in large animal studies of stroke

In 2007, Furuse and colleagues first evaluated IA-CSI in large animals. Infusion into the common carotid artery of six adult canines using a 4-French angiographic catheter rapidly achieved target hypothermic temperatures in target brain parenchyma [51]. Ipsilateral brain tissue was reduced to 33.6 °C by continuous infusion of ringer's solution (6.5 °C) at a rate of 3 ml/kg/min for 30 minutes. Non-negligible changes in rectal temperature, hemoglobin, and hematocrit were observed, likely due to large infusion volumes, but post-procedure histological examination did not reveal any new infarction or hemorrhage[51].

IA-CSI has also been evaluated in rhesus monkeys [52]. Infusion volumes were calculated in proportion to the infusion volumes and body weights of the rats used in the early Ding papers, resulting in a total infusion volume of 100 ml. When infused by IA-CSI at 5 ml/min, 0-4 °C lactated ringer’s solution achieved mild cerebral hypothermia (< 35 °C) within 10 minutes; far faster than systemic infusion using the parameters. Importantly, no significant fluctuations in rectal temperature, hematocrit, cerebral blood velocity, or cerebrovascular reactivity were observed during or after the procedure. Moreover, no cerebral edema, new infarction, hemorrhage, or vasospasm was appreciated, which further validated the safety, feasibility, and efficiency of IA-CSI [52].

A recent study by Caroff and colleagues was one of the first large animal studies to evaluate the optimal flow rate to balance cooling rate with judicious fluid administration [53]. In a canine model, infusion of cold saline (4.5 °C) via the internal carotid artery at 22 ml/min for 25 minutes most effectively cooled the ipsilateral hemisphere while minimizing infusion volume. The optimized infusion parameters were then evaluated for neuroprotective efficacy and found to decrease infarct volumes by an order of magnitude compared to canines that did not receive infusion. An innovative insulated catheter was also employed to minimize heat transfer to the infusion fluid within catheter [53]. The study featured a very small sample size, but nonetheless provided encouraging evidence that IA-CSI can feasibly be performed in large mammals using realistic infusion rates and volumes.

Methods of local hypothermia induction using techniques other than IA-CSI have also been evaluated in several recent large animal studies. A pair of 2015 and 2016 papers evaluated the viability of a unique heat exchanger in a sheep model of cerebral ischemia. The heat exchanger was a novel closed-loop balloon cooling system, similar to the Zoll catheter [54] used in post-cardiac arrest hypothermia [55, 56], which circulates chilled fluids through a long endovascular balloon, thereby cooling passing blood by conduction. The device was optimized for use in the internal carotid artery, and the “core” of the balloon was an endovascular catheter through which mechanical thrombectomy could be performed [55, 56]. While the device was only able to cool blood by approximately 1.5 °C, this endeavor underlines the potential for innovation in that local cooling provides.

Mattingly and colleagues evaluated another alternative cooling method in a tMCAO swine model in 2016 [57]. Using an aneurysm clip, ischemia was induced for 3 hours in 28 pigs. When the clip was released, an outflow catheter (Thermopeutix TwinFlo) was placed in the thoracic aorta, allowing blood to be removed from the body, chilled, then perfused via an inflow catheter placed in the common carotid artery, which was balloon occluded from systemic circulation. Using this technique, target temperatures (< 30 °C) were achieved in a mean time of 15 minutes, and subsequent imaging verified that hypothermic blood perfusion decreased infarct volumes [57]. Furthermore, this extracorporeal system featured a working port, allowing mechanical thrombectomy to be performed through the inflow catheter, much like the device used in the above-stated sheep study.

## IA-CSI in clinical studies of stroke

Given the rousing success of IA-CSI in benchtop research, several pilot human studies have recently been launched. The first group to assess the safety and feasibility of IA-CSI in humans was Choi and colleagues in 2010. This pilot study enrolled 18 patients with partially or completely treated cerebrovascular disease undergoing elective diagnostic cerebral angiographies [58]. Upon completion of the angiogram, the catheter was navigated to the extracranial segment of the internal carotid artery, and cold saline (4-17 °C) was infused at 33 ml/min for 10 minutes. A decrease in jugular venous bulb temperature, a surrogate for brain temperature, was appreciated within seconds of saline infusion, and over the 10-minute trials, bulb temperature decreased by an average of 0.84 °C. In comparison, core body temperature decreased by an average of 0.15 °C. Most importantly, no vital sign derangements were appreciated at any time during the trials. The major drawback of this study was the inability to measure brain temperature directly. In animal studies, thermocouple probes are placed throughout the brain parenchyma for thermal monitoring, but for obvious reasons, this is not feasible in humans. The group acknowledged that, since the jugular venous bulb drains blood from the entire head, the decrease in target parenchymal tissue was likely much greater than the measured 0.84 °C [59, 60]. However, a 2013 follow-up study using a series of previously established biophysical mathematical models estimated that the actual temperature change in the brain parenchyma was approximately 2 °C [61].

The first implementation of IA-CSI in AIS patients was performed in a small 2016 observational study [62]. This non-randomized, single-arm trial enrolled 26 patients with proximal large vessel occlusions between ages 18 and 80 with NIHSS scores ≥ 8 who were eligible for recanalization within 8 hours of symptom onset. In contrast to the infusion protocol used by the Choi’s group, this study performed IA-CSI in two steps. Prior to thrombectomy, 50 ml of chilled saline was infused over 5 minutes via a microcatheter placed through the thrombus. Following thrombectomy, 300 ml of chilled saline was infused into the recanalized vessel over 10 minutes. The former step primarily served to flush accumulated biochemical byproducts from microvasculature in the ischemic region, while the latter step established and maintained cerebral hypothermia. All patients successfully completed the procedure, and no significant changes were appreciated in any of the parameters monitored (rectal temperature, vital signs, electrolytes, or hematocrit). Although the study did not measure venous outflow temperatures, the combination of pre-reperfusion and post-reperfusion IA-CSI was estimated to decrease the temperature in ischemic brain tissue by at least 2 °C, based on the modeling used in the 2013 Choi paper [62]. This study was a milestone for neuroprotection; never before had IA-CSI been performed in AIS patients. As such, the successful completion of the trial was an exciting first step toward better outcomes for the ischemic stroke patient.

Following the success of the 2016 trial, a larger prospective cohort study was designed to explore the safety and efficacy of IA-CSI in patients undergoing mechanical thrombectomy compared to mechanical thrombectomy without IA-CSI [63]. Using the same pre- and post-reperfusion infusion protocol, IA-CSI reduced infarct volumes by an average of 19.1 ml, based on non-contrast CT 3-7 days after surgery. Cold infusion also increased the proportion of patients achieving functional independence by 90 days after stroke (IA-CSI group 51.1% vs control group 41.2%, P=0.192), but this difference was not statistically significant. However, patients in the IA-CSI group had lower Alberta Stroke Program Early Computed Tomography Scores and worse collateral circulation at baseline, so the IA-CSI-provided neurologic function retention is likely more robust than this study indicated [63]. It must be emphasized that, since IA-CSI only conferred statistically significant differences in radiologic infarct size but not neurologic function, these results must be interpreted with caution until the large-scale randomized clinical trial has been published.

It is essential to note that infusion volume optimization has not been evaluated in humans. A pilot study in healthy, conscious volunteers showed that a systemic 30 ml/kg cold saline bolus could be well tolerated without any significant hemodynamic consequences [64]. However, infusion volumes could cause hemodilution, not to mention fluid overload with possible pulmonary edema [53]. As such, IA-CSI should be implemented carefully with a full understanding of the patient’s general condition.

Currently, another exploratory study (UMIN Clinical Trials Registry: UMIN000018255) [65] and a well-designed randomized clinical trial (ClinicalTrials.gov number: NCT03163459) [63] to further evaluate the efficacy of IA-CSI in conjunction with mechanical thrombectomy in AIS patients are ongoing.

## Future direction

There now exists a robust body of preclinical data suggesting that therapeutic hypothermia provides a neuroprotective benefit to the ischemic brain, and that quicker onsets and longer durations enhance this neuroprotection. However, both variables share a common solution: higher infusion rates. As such, a major obstacle in the quest toward IA-CSI clinical implementation is the volume issue; how can we achieve maximally effective cooling while avoiding fluid overload? Here, we propose two possible solutions.

The first solution is to improve hypothermic efficiency. While infusion temperature is often discussed in literature, infusion temperature at the catheter tip is rarely discussed. According to a prospective cohort study, cold saline (4 °C) infused at 10 ml/min through a femoral artery sheath heats to 20.2 °C when it reaches the internal carotid artery [63]. Increased flow rates would minimize heat transfer but are limited by catheter diameter and overall infusion volume. Advances in material sciences may provide solutions. As demonstrated in the Caroff study [53], implementation of innovative catheter designs allows minimization of heat transfer to the greatest extent possible. According to the comparison from the Caroff group, with the same flow rate, saline temperature at the distal tip of their novel insulated catheter was only half of that at the conventional catheter tip, and this cold retention maximizes cooling rate.

The second solution is to improve the method of IA-CSI. Several studies have evaluated the efficacy of hypothermic autologous arterial blood as the perfusate, which eliminates the concern for fluid overload, thereby alleviating any restriction on hypothermia duration. Previous pre-clinical studies have assessed this modified IA-CSI approach in rodents and large animals [66-68]. In these studies, the unilateral internal carotid artery and the ipsilateral femoral artery were occlusively cannulated with a catheter joined to an extracorporeal centrifugal pump with cooling function [67, 68]. Driven by the centrifugal pump, femoral arterial blood was continuously withdrawn, cooled, and infused into the internal carotid artery to induce selective brain cooling [67, 68]. The Mattingly study reviewed above modified this technique by placing the outflow catheter in the descending thoracic aorta [57]. All of these investigations have found this method to rapidly lower brain temperatures, reduce infarct volumes, and improve neurologic function in test subjects without hemodilution [66, 68]. This technique was also tentatively utilized in a human patient with a massive aneurysm without any reported adverse events [69]. A major issue with the technique is the need for liberal anticoagulation, which predisposes to catastrophic outcomes in the case of ischemic to hemorrhagic transformation. However, further investigations can likely optimize anticoagulation to minimize bleeding risk. Given the infancy of this concept, it is likely that a much more effective, streamlined local autologous blood cooling method could be established, which may serve as the ultimate solution to the cooling problem.

## Conclusion

After nearly 20 years of unrelenting efforts, the neuroprotective effects of IA-CSI in AIS have been gradually verified in rodent studies, large animal studies, and now in clinical investigations. Although the clinical study of IA-CSI in AIS is only just beginning, with the progress of medical technology and the development of new materials, IA-CSI will continue to become more effective and more attractive. Therapeutic local hypothermia, represented by IA-CSI, is ushering in a development opportunity, and as it gains momentum, clinical acceptance will likely follow.
